# Isolated COVID-19 Infection Precipitates Myasthenia Gravis Crisis: A Case Report

**DOI:** 10.5811/cpcem.2020.9.49049

**Published:** 2020-10-09

**Authors:** Phillip Moschella, Prerana Roth

**Affiliations:** * University of South Carolina School of Medicine Greenville, Prisma Health: Upstate Affiliate, Department of Emergency Medicine, Greenville, South Carolina; † University of South Carolina School of Medicine Greenville, Prisma Health: Upstate Affiliate, Department of Infectious Disease, Greenville, South Carolina

**Keywords:** COVID-19, plasma exchange, myasthenic crisis

## Abstract

**Introduction:**

Coronavirus disease 2019 (COVID-19) has spread around the world and caused hundreds of thousands of fatalities across a wide spectrum of patients with varying severity and presenting complaints. The discussion of the ability of this disease to cause significant illness in patients with various risk factors such as myasthenia gravis is important to help guide physicians on recognition and treatment options as the pandemic matures.

**Case Report:**

Here we discuss a single case of isolated COVID-19 infection that precipitated a myasthenic crisis with no other clinical sequelae in a patient who presented to the emergency department (ED). This report highlights some of the initial difficulties and delay in diagnosis encountered earlier in the pandemic with limited testing supplies and processing labs; however, prompt ED recognition and treatment still led to a favorable outcome.

**Conclusion:**

The patient recovered during this initial presentation and was successfully treated with plasma exchange and steroids only. It is important to recognize that myasthenia gravis patients may represent a uniquely vulnerable population that requires enhanced surveillance and screening to prevent significant morbidity and mortality. This case describes how even a mild infection with no significant clinical sequelae or significant signs on imaging studied can precipitate a crisis event.

## INTRODUCTION

The current disease outbreak featuring the novel coronavirus (severe acute respiratory syndrome coronavirus; SARS-CoV-2) has sparked a global pandemic, namely coronavirus disease 2019 (COVID-19). This novel coronavirus belongs to the *Coronaviridae* family and is comprised of single-stranded, positive-sense ribonucleic acid genomes that can cause both respiratory and/or enteric disease symptoms.^1^ The current SARS-CoV-2 pandemic started in China in late 2019 and has sickened millions and killed thousands across the globe.^2^,^3^, COVID-19 primarily causes a respiratory disease with a wide spectrum of disease severity that ranges from mild to almost no upper respiratory symptoms to severe acute respiratory distress syndrome, pneumonia, multiorgan failure, and death.^2^ The world’s experience with the SARS-CoV-2 pandemic is rapidly evolving as the virus continues to spread with increased mortality in high-risk groups of patients, including the elderly and those with comorbidities such as obesity and diabetes.

It is not yet known how COVID-19 affects myasthenia gravis patients. To date, there are two published reports of only six total patients that have been described in the current literature.^4^,^5^ These isolated reports provide only limited guidance for the emergency physician on the recognition of COVID-19 in myasthenia gravis patients as they may present to the emergency department (ED), and none describe successful treatment using plasma exchange. This report highlights how isolated infection with COVID-19 with no signs on initial imaging studies can trigger a myasthenic crisis without any other clinical symptoms and how this patient was successfully treated with plasma exchange and steroid therapy alone.

## CASE REPORT

A 70-year-old male with a history of myasthenia gravis, hypertension, hyperlipidemia, diabetes, and coronary artery disease presented to one of our EDs in March 2020 with shortness of breath and cough. While he did not routinely use supplemental oxygen at home, he had started using it recently as he felt he was having a myasthenic crisis. The patient denied chest pain or fevers but did report some subjective chills and a non-productive cough. Overall, he described that he felt as though his chest was “not moving well and it’s weak” and that he was “belly breathing.” The patient reported that he was strictly adhering to social distancing and the stay-at-home recommendations because of his health history and had no known sick contacts, no recent travel, no new medications, and was not on any antibiotic therapy.

The patient’s vital signs in triage were as follows: heart rate 68 beats per minute; blood pressure 99/51 millimeters of mercury (mm Hg), respiration rate 18 breaths per minute; temperature: 36.3° Celsius; and with an oxygen saturation 96% on two liters nasal cannula as he could not tolerate room air alone. His physical exam revealed a respiratory rate of 28 breaths per minute. He was only able to speak in two-word sentences and had labored breathing with diminished breath sounds throughout. After taking a deep breath, he was only able to get to the letter “D” when reciting the alphabet. Evaluation by respiratory therapy showed a negative inspiratory force of −10 mm Hg (reference (ref) range: >−60 mm Hg) and a vital capacity of 960 milliliters (mL) (ref range: >30mL/kilogram ideal body weight). There were no other significant findings on physical exam.

Initial venous blood gas on two liters nasal cannula showed partial pressure of oxygen of 37 mm Hg (30–40 mm Hg), and partial pressure of carbon dioxide of 53 mm Hg (ref range: 35–45 mm Hg) with a pH of 7.4 (ref range: 7.35–7.45). Other laboratory work-up included the following: complete blood count; comprehensive metabolic panel; B-type natriuretic peptide (BNP), lactic acid; two blood cultures; procalcitonin; serum cortisol level; thyroid stimulating hormone; and free thyroxine levels. The patient had a mild leukocytosis with a white blood cell count of 12.5 × 10^9^/liter (L) (ref range: 4.5 to 11.0 × 10^9^/L). His procalcitonin was negative at 0.02 nanograms (ng)/mL (<0.15 ng/mL), and brain-type natriuretic peptide only mildly elevated at 147 picograms (pg)/mL (ref range: <100 pg/mL). His initial troponin was 0.07 ng/mL (ref range: <0.1 ng/mL). Renal function was normal with a creatinine of 0.74 milligrams per deciliter (mg/dL) (ref range: 0.6–1.2 mg/dL).

CPC-EM Capsule

What do we already know about this clinical entity?

*Myasthenia gravis carries the risk of significant morbidity and mortality. It is unknown whether coronavirus disease 2019 (COVID-19) infection can precipitate a crisis*.

What makes this presentation of disease reportable?

*This is one of only a few reports demonstrating an association between COVID-19 infection and myasthenic crises*.

What is the major learning point?

*COVID-19 can present atypically in these patients and and likely precipitate a myasthenic crisis. This patient was successfully treated with steroids and plasma exchange*.

How might this improve emergency medicine practice?

*Providers should include surveillance for COVID-19 infection in patients with myasthenia gravis to prevent significant morbidity or mortality*.

His admission chest radiograph (CXR) showed no acute cardiopulmonary abnormality. His other laboratory values were unremarkable. A SARS-CoV-2 polymerase chain reaction (PCR) test was sent to the health department as per hospital protocol. At that time, our hospital policy was to add routine testing for all patients admitted for any respiratory complaints to help guide personal protective equipment use and transfer positive patients to designated COVID-19 hospitals within our system. The result of this testing was pending at this time of initial admission.

Due to impending respiratory compromise, and following discussion with the patient and his wife, it was decided to proceed with intubation in the ED. The initial diagnosis was acute myasthenia gravis crisis. The patient’s myasthenia gravis had been diagnosed in November 2019 when he presented with stroke-like symptoms of diplopia and dysphagia. He was found to have acetycholine receptor antibody positive/Musk-receptor antibody negative myasthenia gravis. Since his initial diagnosis in 2019, he had been well controlled on prednisone 30 mg daily, methotrexate, and pyrostigmine 60 mg four times daily. He had no hospitalizations between discharge in December 2019 and this case presentation in March 2020.

Due to persistent hypotension during this encounter, stress-dose steroids (100 mg of hydrocortisone sodium succinate) were started in the ED. Neurology recommended immediately starting plasma exchange, and the patient underwent five sessions in the ensuing five days. The SARS-CoV-2 PCR resulted as positive on day three, and infectious disease was consulted. The risks of medication interactions with hydroxychloroquine was thought to outweigh the benefits and thus was not initiated. The patient was not a candidate for remdesivir as part of an initial medication trial as he never developed any infiltrates on CXR throughout his hospital course. Current data is evolving but early reports show only a 68% sensitivity for CXR in diagnosing COVID-19 at time of admission.^6^ Thus, in total no specific COVID-19 treatments were pursued. Interestingly, with plasma exchange and stress-dose steroid therapy alone, the patient was able to be extubated on day five and had no other complications during his hospital course through discharge.

## DISCUSSION

Knowledge regarding how SARS-CoV-2 affects different populations is accumulating as researchers describe their experiences during this pandemic. There are a few case reports of COVID-19 causing myasthenic crises; however, the outcomes and disease course seem to vary widely.^4^,^5^ It is unclear which patient or disease feature can result in worse outcomes, and thus more study is needed. Current treatment guidelines for myasthenic crises include both intravenous immunoglobulin (IVIG) and plasma exchange as first-line options.^7^ In this particular situation of COVID-19 triggering a crisis we believe that using plasma exchange expedited recovery through removing inflammatory cytokines related to COVID-19 infection that may have been one of the triggers for this patient’s myasthenic crisis. Studies are ongoing to assess the efficacy of plasma exchange as a treatment for COVID-19. Currently available IVIG would not have protective antibodies against COVID-19; thus, therapeutic plasma exchange was preferable over IVIG in this particular situation.

## CONCLUSION

This case highlights that COVID-19 can present atypically in patients with myasthenia gravis and can independently precipitate a crisis event. In addition, judicious but prompt intubation, stress-dose steroids, and plasma exchange may be integral in the treatment of patients with myasthenia gravis and COVID-19.
